# Time-Restricted Feeding Improves Body Weight Gain, Lipid Profiles, and Atherogenic Indices in Cafeteria-Diet-Fed Rats: Role of Browning of Inguinal White Adipose Tissue

**DOI:** 10.3390/nu12082185

**Published:** 2020-07-23

**Authors:** Samira Aouichat, Meriem Chayah, Souhila Bouguerra-Aouichat, Ahmad Agil

**Affiliations:** 1 Department of Pharmacology, Biohealth Institute and Neuroscience Institute, School of Medicine, University of Granada, 18016 Granada, Spain; samira_aouichat@outlook.fr (S.A.); meriem.chyah@gmail.com (M.C.); 2 Team of Cellular and Molecular Physiopathology, Faculty of Biological Sciences, University of Sciences and Technology Houari Boumediene, El Alia, 16011 Algiers, Algeria; souhila.aouichat@hotmail.fr

**Keywords:** time-restricted feeding, cafeteria diet, obesity, lipid profiles, atherogenic indices, white adipose tissue browning

## Abstract

Time-restricted feeding (TRF) showed a potent effect in preventing obesity and improving metabolicoutcomes in several animal models of obesity. However, there is, as of yet, scarce evidence concerning its effectiveness against obesogenic challenges that more accurately mimic human Western diets, such as the cafeteria diet. Moreover, the mechanism for its efficacy is poorly understood. White adipose browning has been linked to body weight loss. Herein, we tested whether TRF has the potential to induce browning of inguinal white adipose tissue (iWAT) and to attenuate obesity and associated dyslipidemia in a cafeteria-diet-induced obesity model. Male Wistar rats were fed normal laboratory chow (NC) or cafeteria diet (CAF) for 16 weeks and were subdivided into two groups that were subjected to either ad libitum (ad lib, A) or TRF (R) for 8 h per day. Rats under the TRF regimen had a lower body weight gain and adiposity than the diet-matchedad lib rats, despite equivalent levels of food intake and locomotor activity. In addition, TRF improved the deranged lipid profile (total cholesterol (TC), triglycerides (TG), high-density lipoprotein (HDL-c), low-density lipoprotein (LDL-c)) and atherogenic indices (atherogenic index of plasma (AIP), atherogenic coefficient (AC), coronary risk index (CRI) in CAF-fed rats. Remarkably, TRF resulted in decreased size of adipocytes and induced emergence of multilocular brown-like adipocytes in iWAT of NC- and CAF-fed rats. Protein expression of browning markers, such as uncoupling protein-1 (UCP1) and peroxisome proliferator-activated receptor gamma coactivator 1-alpha (PGC1α), were also up-regulated in the iWAToftime-restricted NC- or CAF-fed rats. These findings suggest that a TRF regimen is an effective strategy to improve CAF diet-induced obesity, probably via a mechanismthe involving WAT browning process.

## 1. Introduction

Obesity is defined as abnormal or excessive fat accumulation that may impair health [1]. The etiology of obesity is quite complicated owing to the involvement of both genetic and environmental factors. Among the environmental factors, diet plays a significant role in the development of obesity [2]. Modern nutritional patterns, termed as “Western diets”, including high-fat and -cholesterol, high-protein, high-sugar, and excess salt intake, as well as frequent consumption of processed and fast foods, cause a high prevalence of obesity in Western societies [3,4]. According to the data published by the World Health Organization, the worldwide prevalence of obesity increased threefold over the last 40 year sand is expected to reach ~30.3 billion by 2030 [1]. Obesity is often associated with a condition known as metabolic syndrome and characterized by insulin resistance, dyslipidemia, hypertension, and systemic inflammation, as well as cardiovascular diseases [5]. Classical attempts to develop therapeutic strategies have mostly focused on limiting caloric intake by diet and increasing energy expenditure by physical exercise; however, these strategies are often not effective, withunsatisfactory results and success limited to a small percentage of individuals [6]. Today, TRF, an eating pattern that limits food intake to a specific number of hours (≤12 h) without altering nutrient quality or quantity, is emerging as a promising therapeutic strategy against obesity and associated metabolic disorders [7]. One outstanding study pioneered by Panda’s groupdemonstrated that mice fed a high-fat diet (HFD) under TRF paradigm of 8 h per day during the active phase were protected against obesity, hyperinsulinemia, hepatic steatosis, and inflammation, even though they consume equivalent calories to those with ad libaccess to the HFD [8]. Interestingly, another study by the same group showed thata TRF of 8–12 h without reducing caloric intake in mice prevented and even reversed obesity and related metabolic disorders arising from a variety of obesogenic challenges, including high-fructose and high-fat, high-sucrose diets [9]. To date, there have been extensive studies in both rodents and humans reporting that TRF reduces body weight, improves glycemic control and insulin resistance, prevents atherogenic dyslipidemia, reduces blood pressure, prevents hepatic steatosis, and improves inflammatory markers in diet-inducedobesity models (DIO) [10,11,12,13,14,15,16,17,18,19,20,21,22,23,24,25]. It should be noted that most rodent studies have attempted to mimic the unhealthy human Western diet through the use of numerous obesogenic diets, which generally focused on a single or a combined macronutrient (s), in particular, fat and/or sugar. However, these commercial high-fat, high-sugar or high-fat, high sugar rodent diets are not analogous to the highly processed food common in Western societies and associated with increased global obesity rates. The closest comparable to human Western foods is the cafeteria diet (CAF), as it provides animals with nutritionally varied diet of high-energy, palatable human foods that are low in fiber and contain substantial amounts of fat, sugar, and salt (for example, cheese, chocolate, processed meat, chips, and cookies), thereby recapitulating the key obesogenic features of the unhealthy human diet [26,27]. Furthermore, an extended CAFdiet in rodents produced an exaggerated phenotype of obesity with excessive body weight gain, pronounced adiposity, dyslipidemia, and liver inflammation [28,29,30] and has been argued to model modern human obesity and related metabolic disorders more severely than the classical HFD mode [28]. In addition, a CAFdiet exerted a robust impact upon appetite and energy intake due to its high amount of sugar, salt, and additives, such as appetizers and flavor enhancers, compared to the traditional HFD [31]. To the best of our knowledge, to date no study has reported on whether a TRF has the potential to prevent obesity and related metabolic disarrangements in a CAF-diet-induced obesity model. Furthermore, the mechanism that underlies the beneficial effect of the TRF paradigm on obesity is still not welldefined.Various mechanisms have been proposed to explain the anti-obesity effect of a TRF regimen, includingentraining the circadian clock to a fixed feeding time [32], altering gut microbiome [33], and increasing free fatty acid mobilization and fat oxidation [8]. However, it remains to be determined whether TRF can trigger white adipose tissue (WAT) browning. WAT browning, a process of formation of brown-like adipocytes within WAT, is regarded as a potential therapeutic target for obesity due to its unique capacity to up-regulate thermogenesis and thusincrease energy expenditure through glucose and fatty acidoxidation.Brown-like adipocytes, termed “beige cells”, are characterized by multilocular lipid droplets and high amounts of UCP1-positive mitochondria, and they switch from an energy storage to an energy expenditure state by expressing thermogenic markers, including UCP1 and PGC1α, which constitute the molecular signature of brown and beige adipocytes and play an important role in metabolic thermogenesis [34]. Classically, browning of WAT can be induced in animals and humans by various physiological cues, such as exercise and cold, and pharmacological agents, suchasan active ingredient like melatonin, which has previously been demonstrated by our group to have an iWAT browning potential in obese diabetic animals [35]. Based on previous findings showing that TRF regimen reduced body weight gain in DOI without altering caloric intake or activity level [8,12,19], we hypothesized that a TRF regimen would prevent obesity via promoting WAT browning. Hence, the present study was aimed to address whethera TRF schedule of 8 h per day during the active phase can attenuate excessive body weight gain inCAF-diet-induced obeserats. There arescarce data available in the literature regarding the effects of TRF on lipid profile and atherogenicity, in animal models of obesity, and therefore, we determined whether TRF can prevent the dyslipidemia produced in response to a CAFdiet. Additionally, the potential role of TRF paradigm as a white fat browning inducer was also explored.

## 2. Materials and Methods

### 2.1. Animals

All research and animal care procedures were authorized and approved by the Institutional Animal Care Committee of the National Administration of Algerian Higher Education and Scientific Research (Ethical approval number: 981-1 law of 22 August 1998).

A total of 24 male albino Wistar rats (*Rattusnorvegicus*) weighing 120–130 g were obtained from the Pasteur Institute of Algiers, Algeria. Rats were single-housed in polypropylene cages with a stainless steel lid under thermoneutrality conditions (28–30 °C) and a 12 h light: 12 h dark schedule, with lights on at 08:00 a.m. (Zeitgeber time 0, ZT0).Normal rat chow pellets (supplied by ONAB, Algiers) and water were provided ad lib. All animals were acclimatized to their new environment for 15 days before starting the experiment.

### 2.2. Experimental Feeding Schedule and Diets

**Figure 1:**
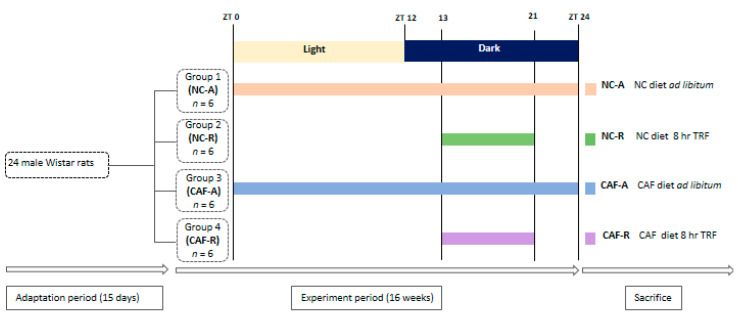
Schematic representation of the experimental feeding schedule. Male Wistar rats were allowed a 15-dayadaptationperiod prior to beginning the experiment and were divided into four groups as follows: NC–A (normal chow, ad libfeeding); NC–R (normal chow time, restricted feeding); CAF–A (cafeteria diet, ad libfeeding); and CAF–R (cafeteria diet, time-restricted feeding). Time-restricted feeding groups were allowed to feed for 8 h per day during the dark phase between ZT 13 and ZT 21. After sixteen weeks of experiment, animals were sacrificed. CAF: cafeteria diet; NC: normal chow diet; TRF: time-restricted feeding; ZT: Zeitgeber.

Upon initiation of the experiment, rats (initial body weight 150 ± 1.5 g) were randomized by weight into four groups (*n* = 6 per group) to ensure equivalent starting body weight. They were maintained on the normal chow diet (NC) or switched to a pre-selection of highly palatable, energy-dense human foods consisting of cookies, cereals, chocolate, crackers, chips, cheese, processed meat, etc. (cafeteria diet; CAF), and they were subdivided into two groups per diet.One group/diet had ad libitum (ad lib; A) access to food: NC–A (*n* = 6) and CAF–A (*n* = 6) rats. The second group/diet had temporal restriction (R) to food: NC–R (*n* = 6) and CAF–R (*n* = 6) rats. These feeding regimens continued for 16 wks until the end of the experiment.Rats under the TRF regimen were allowed access to the diet for 8 h per day during the active phase (dark period) from ZT 13 (1 h after lights off) to ZT 21 (3 h before lights on). Food access was monitored by manually switching the rats from cages with food and water to cages with only water, to avoid foraging and coprophagia. Rats fed ad lib were also transferred between feeding cages at the same time to standardize handling stress and minimize experimental variation between groups.The CAF-diet protocol used in this study was adapted from a previous study [28] and consisted of 19 different highly palatable and energy-rich unhealthy human snack foods purchased from a supermarket in Algeria, accompanied by normal rat chow pallets. The list of cafeteria diet food items with nutrient compositions is listed in Supplementary Table S1. The normal chow diet is composed of whole ground corn, soybean meal, and wheat bran and is supplemented with minerals, amino acids, and vitamins and had an energy density of 310 Kcal/100 g (56% carbohydrate, 24% protein, and 16% lipid), whereasthe CAFdiet had an energy density (taken from the daily offering of each component) of 501 Kcal/100 g on average (37% carbohydrate, 16% protein, and 47% lipid). Three different items plus normal rat chow pallets were provided daily in excess quantities to each animal. To maintain variety and thus induce sustained hyperphagia, the food items provided were altered daily by replacing the three food items with new fresh ones. The menu was varied daily in a manner that ensuresa similar proportion of fat, protein, sugar, and carbohydrate in each daily set of CAF foods. Normal chow-diet and CAF-diet food items were pre-weighed individually and presented to the rats at ZT 13, and the leftoverswere weighed after 24 and 8 h in ad liband time-restrictedfed rats, respectively. To carefully monitor the CAF food consumption, food spillage was meticulously collected from the cage floor, and the difference between presented and recovered food items was corrected for drying, as previously described by Shafat et al. [31]. Calorie intake was calculated as weekly intake, based on nutritional information provided by the manufacturer (Supplementary Table S1). The body weight was recorded weekly over 16 wks between 09:00 and 10:00 a.m. The experimental protocol is described in Figure 1.

### 2.3. Locomotor Activity Assessment

At week fifteen, the locomotor activity was evaluated in the open field by counting the number of squares crossing and occurrences of rearing behavior.Those two variables are indicative of general activity levels [36]. The test was performed in a silent room at night, 2 h after light off (ZT 14), in a four-sided 90 × 90 × 45 cm varnished wooden box, and the floor of the open field was divided into 25 equal-size squares (18 cm). In order to avoid any potential food anticipatory activity, which was reported to occur a few hours prior the arrival of food [37], we first started to assess the locomotor activity in ad lib groups, followedby TRF groups. Data from all groups of rats were collected within approximately 2 h (between ZT 14 and ZT 16). Each rat was placed singly in the center of the open field and given 5 min to explore. A rat was recorded as crossing a square when more than three paws or half of the body crossed the boundary into the nearby squares. Rearing was defined from the moment when both forelimbs raised at least 1 cm above the floor. At the end of each trial, the box was cleaned with water and wiped dry before introducing the next animal [38].

### 2.4. Blood and Tissue Collection

At the end of the experimental period, rats were fasted for ~16 h starting from ZT 21, anesthetized by urethane (1 g/kg b.w, i.p.), and scarified. Bloodwas collected via heart puncture, and serum was obtained by centrifugation at 3000 r.p.m for 15 min at 4 °C. The recovered serum was keptfrozen at −20 °C until analysis. White adipose tissue depots from mesenteric (mWAT), epididymal (eWAT), retroperitoneal (rWAT), and inguinal subcutaneous (iWAT) sites were rapidly removed, rinsed with phosphate buffer (PBS (phosphate-buffered saline), and then weighed, followed by immediate freezing in liquid nitrogen. Serum and iWAT specimens were shipped on dry ice to the laboratory of Prof. Ahmad Agil (UGR, Spain) and then stored at −80 °C for lipid profiles and Western blot analysis.

### 2.5. Measurement of Lipid Profiles and Atherogenic Indices

Serum levels of triglycerides (TG), total cholesterol (TC), high-density lipoprotein cholesterol (HDL-c), and low-density lipoprotein cholesterol (LDL-c) were measured using commercial kits in an automatic analyzer (Roche-Hitani molecular system 902, Branchburg, NJ, USA).

To predict atherosclerosis and cardiovascular problems, the atherogenic indices (atherogenic index of plasma (AIP); atherogenic coefficient (AC); coronary risk index (CRI)) were calculated using the following equations [39,40,41]:

AIP = Log_10_ [TG/HDL-c]

AC = [TC − HDL-c/HDL-c]

CRI = [TC/HDL-c]

### 2.6. Microscopic Analysis

Following anesthesia, excised inguinal fat was fixed by immersion in 4% formaldehyde in 0.1 M phosphate buffer overnight at 4 °C, washed inPBS, dehydrated in a graded series of ethanol, cleared in xylene, and embedded in paraffin. The paraffin blocks from each group were cut with a microtome into 5 µm thick sections, stained with hematoxylin and eosin (H&E), and inspected under a light microscope (×400, Olympus, Germany) equipped with a digital camera system (Carl Zeiss camera, model Axiocam ERc 5s. Germany). Images of H&E-stained tissue sections were digitized, and adipocyte size and percentage of multilocularity were determined usingAxio Vision software (Carl Zeiss Imaging Solutions). The average adipocyte size was expressed as the average cross-sectional area per cell (μm^2^/cell) of tissue sample, which was calculated based on the values of at least 20 adipocytes in 10 random fields per section. The percentage of total adipocyte population showing multilocularity was determined by quantifying the area of multilocular adipocytes in relation to the entire area of the section. Histological slides were performed and examined by a technician who was blinded to tissue group identity.

### 2.7. Western Blotting Analysis

About 100–200 mg of iWAT was homogenized in lysis buffer (150 mmol NaCl, 5 mmol EDTA, 50 mmol Tris-HCl, and pH 7.4) without Triton X-100 and homogenized with a Teflon pestle. Homogenates were centrifuged (3000× *g* for 15 min, 4 °C), and the fat cake was removed from the top of the tube. Then, Triton X-100 was added to a final concentration of 1%. After incubating at 4 °C for 30 min, extracts were cleared by centrifugation at 15,000× *g* for 15 min at 4 °C. One hundred micrograms of total protein was analyzed by SDS-PAGE (sodium dodecyl sulfate polyacrylamide gel electrophoresis). The gels for immunoblot analyses were transferred to a nitrocellulose membrane (Bio-Rad Trans-Blot SD; Bio-Rad Laboratories, Hercules, CA, USA). The membranes were cut at UCP1 and PGC1α molecular weight level (33 kDa), and blots were reacted with a 1:2000 dilution of anti-UCP1 produced in rabbit (Sigma Aldrich, U6382, St. Louis, MO, USA), in blocking solution (PBS, 5% nonfat milk) and anti-PGC1α produced in rabbit. β-actin antibody generated in mouse (Santa Cruz Biotechnology, SC-81178, Santa Cruz, CA, USA) was used as a control. Horseradish peroxidase labeled secondary antibodies were goat anti-mouse IgG and goat anti-rabbit IgG (1:1000, Sigma Aldrich). Proteins were visualized by enhanced chemiluminescence (ECL kit, GE Healthcare Life Sciences, Pittsburgh, PA, USA).

### 2.8. Statistical Analysis

Statistical Package of Social Science (IBM SPSS Software, version 22) was used for statistical analysis. All results are expressed as mean ± standard deviation (S.D.) values. Two-way ANOVA was used to examine two variables (diet and schedule feeding). When a significant F score on two-way ANOVA was recorded, pair-wise comparisons were conducted using aMann–Whitney U-test. Repeated-measures two-way ANOVA was also used to analysis change in body weight over time. Following a significant result, single time point comparisons were made using unpaired Mann-Whitney U-test. Differences between group means were considered statistically significant if *p* < 0.05.

## 3. Results

### 3.1. Effects of TRF on Body Weight, Calorie Intake, Adipose Weight, and Locomotor Activity

**Figure 2:**
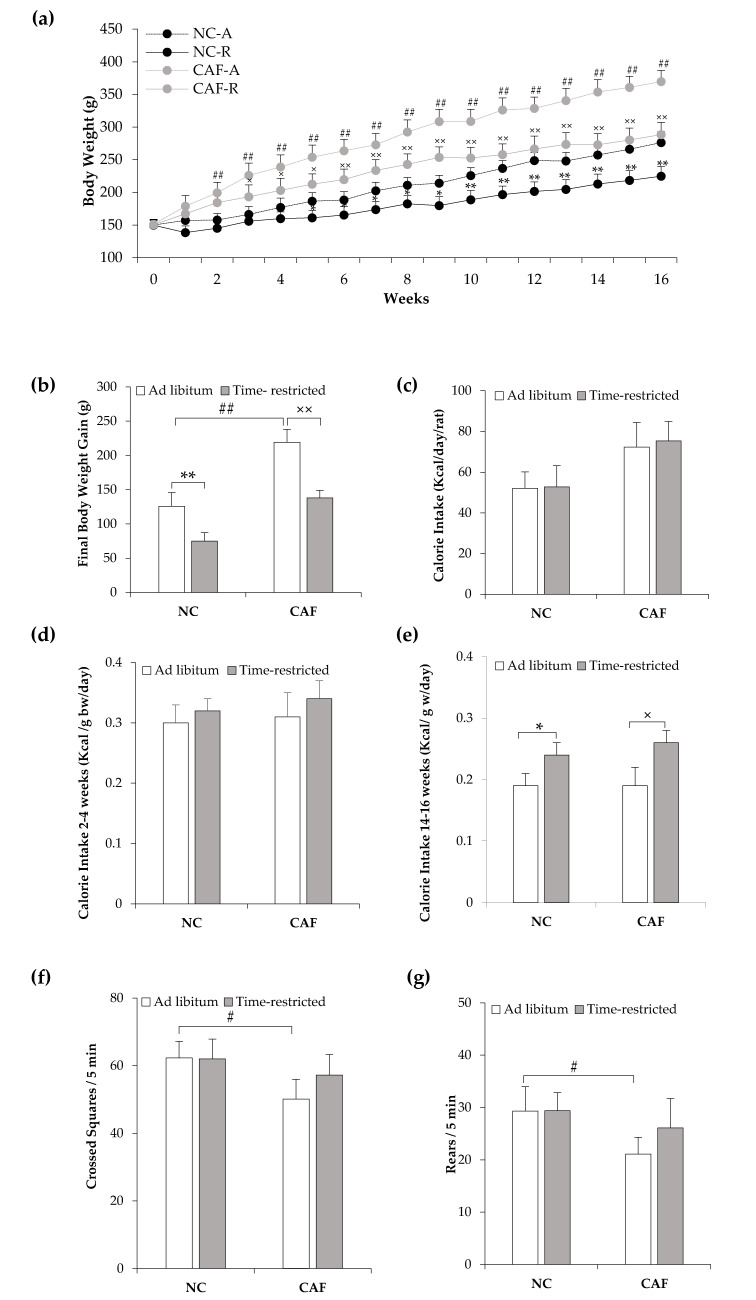
Effects of time-restricted feeding on body weight change, calorie intake, and locomotor activity in normalchow- and cafeteria-fed rats. (**a**) Weekly body weight change over the experiment; (**b**) final body weight gain; (**c**) total daily calorie intake; (**d**,**e**) daily calorie intake normalized to unit body weight: (**d**) weeks 2–4; (**e**) weeks 14–16; (**f**,**g**) locomotor activity on the 15th week of the experiment: (**f**) number of crossing squares; (**g**) number of rears. NC–A: normal chow ad libfeeding; NC–R: normal chow time-restricted feeding; CAF–A: cafeteria diet ad libfeeding; CAF–R: cafeteria time-restricted feeding; NC: normal chow diet; CAF: cafeteria diet; b.w: body weight. Values are means ± S.D. (*n* = 6). ^#^ *p* < 0.05, ^##^ *p* < 0.01 CAF–A vs. NC–A rats; * *p* < 0.05, ** *p* < 0.01 NC–R vs. NC–A rats; ^×^ *p* < 0.05, ^××^ *p* < 0.01 CAF–R vs. CAF–A rats (Mann–Whitney U-test).

To evaluate the effectiveness of a TRF regimen to protect against CAF-diet-induced obesity, we first tested its effect on body weight. As expected, rats fed a CAF-diet on ad libaccess exhibited a significant increase in body weight from the 2nd week of the experiment until the end (16 wks) as compared to rats fed a NCdiet ad lib (*p* < 0.01; Figure 2a). The final body weight gain in the CAF–A group was significantly higher compared with that in the NC–A group (219.7 ± 18.9 g vs. 126.5 ± 13.0 g; *p <* 0.01; Figure 2b). The TRF regimen significantly reduced body weight in rats fed with both NC- and CAF-diets, from respectively the 5th and 3rd week until the end of the experiment (*p* < 0.05; Figure 2a). The final body weight gain in NC–R (75.0 ± 11.0 g) and CAF–R (138.1 ± 12.3 g) groups was significantly lower compared to that in their diet-matched ad lib groups (*p* < 0.01; Figure 2b). Interestingly, the final body weight gain in the CAF–R group was not significantly different from the NC–A group (*p* > 0.05; Figure 2b).

Although the final body weight gain was lower in TRF rats, the daily total calorie intake showed no statistically significant difference between time-restricted and ad lib rats that were fed with either NC or CAFdiet (*p* > 0.05; Figure 2c). Notably, total daily calorie intakes per individual rat were 52.1 ± 8.0, 52.8 ± 10.4, 72.4 ± 12.0, and 75.3 ± 9.6 Kcal in the NC–A, NC–R, CAF–A, and CAF–R groups, respectively.When normalized to body weight, NC–R and CAF–R rats consumed similar calories at the beginning of the experiment (weeks 2–4), as compared to their diet-matched ad lib rats (*p* > 0.05; Figure 2d). Daily calorie intakes per gram body weight was 0.30 ± 0.07, 0.32 ± 0.05, 0.31 ± 0.10, and 0.34 ± 0.07 Kcal in the NC–A, NC–R, CAF–A, and CAF–R groups, respectively. By the end of the experiment (week 14–16), NC–R and CAF–R rats consumed higher calories than their ad libcounterparts (*p* < 0.05; Figure 2e). The values were 0.19 ± 0.05, 0.24 ± 0.05, 0.18 ± 0.07, and 0.26 ± 0.05 Kcal/g body weight/day in the NC–A, NC–R, CAF–A, and CAF–R rats, respectively.

To test whether the reduced body weight gain, in the TRF rats, was due to locomotor activity, animals were tested in an open-field arena to evaluate the spontaneous locomotor activity. Notably, the CAF–A group exhibited reduced locomotor activity compared withthe NC–A group, since the CAF–A group showed an overall decrease in the number of crossing squares (53.1 ± 6.9 vr. 62.3 ± 4.9; *p* < 0.05; Figure 2f) and rears (23.1 ± 7.4 vr. 29.3 ± 4.7; *p* < 0.05; Figure 2g). Importantly, the TRF schedule had no significant effect on locomotor activity. Indeed, the number of crossing squares and rears in NC–R (62.0 ± 5.9 and 29.4 ± 3.4, respectively, Figure 2f,g) and CAF–R (57.2 ± 6.1 and 26.1 ± 5.6, respectively, Figure 2f,g) groups had no significant differences(*p* > 0.05)compared totheir ad lib counterparts, although they tended to be lower in the CAF–A group, compared to the CAF–R group.

To identify whether the TRF paradigm had an effect onspecific adipose depots, we weighed adipose tissues from different anatomical locations. As expected from their body weights, the CAF–A group showed significantly higher total adiposity than the NC–A group (*p* < 0.01; Table 1). The TRF paradigm significantly reduced the weight of the iWAT and all the visceral adipose tissue depots (mesenteric, epididymal, and retroperitoneal) in both NC- and CAF-fed rats (*p* < 0.05; Table 1). The total adiposity is significantly lower in TRFrats compared to that in ad librats (*p* < 0.01; Table 1). Two-way ANOVA revealed a significant main effect for diet on number of crossing squares and rears without intersection of diet and schedule feeding (*p* > 0.05).

### 3.2. Effects of TRF on Lipid Profiles and Atherogenic Indices

To determine whether a TRF regimen could prevent dyslipidemia in the CAF-fed rats, serum lipid profiles and atherogenic indices were evaluated. As shown in Table 2, the CAF–A group showed a significant increase in serum levels of TG, TC, and LDL-c as compared to the NC–A group (*p* < 0.01). After 16 wks of TRF schedule, serum TG, TC, and LDL-c levels in the CAF-fed rats were significantly decreased by approximately 19.8%, 27.8%, and 43.5%, respectively, as compared to those inthe CAF–A group (*p* < 0.01; Table 2). Serum HDL-c levels in the CAF–A group weresignificantly lower than thoseinthe NC–A group (*p* < 0.01; Table 2); however, the TRF paradigm increased serum HDL-c levels by approximately 29.6% ascompared to thoseinthe CAF–A group (*p <* 0.01; Table 2).

Atherogenic and cardioprotective properties of the TRF are also presented in Table 2. The CAF–A group showed significantly higher AIP, AC, and CRI (*p* < 0.01) than the NC–A group. It was interesting to note that the TRF regimen ameliorated these atherogenic indices in CAF-fed rats.Indeed, the values of AIP, AC, and CRI were significantly reduced in the CAF–R group when compared to those recorded for their ad lib counterparts (*p* < 0.01; Table 2), and they were similar to those inthe NC–A group.Nevertheless, the TRF had no significant effectoneither lipid profiles (TG, TC, HDL-c, and LDL-c) oratherogenic indices (AIP, AC, and CRI) inNC-fed rats (*p* > 0.05; Table 2). This is confirmed by two-way ANOVA between groups that showed a significant interaction between schedule feeding and diet on lipid profiles and atherogenic indices (*p* < 0.05).

### 3.3. Effects of TRF on iWAT Morphology

**Figure 3:**
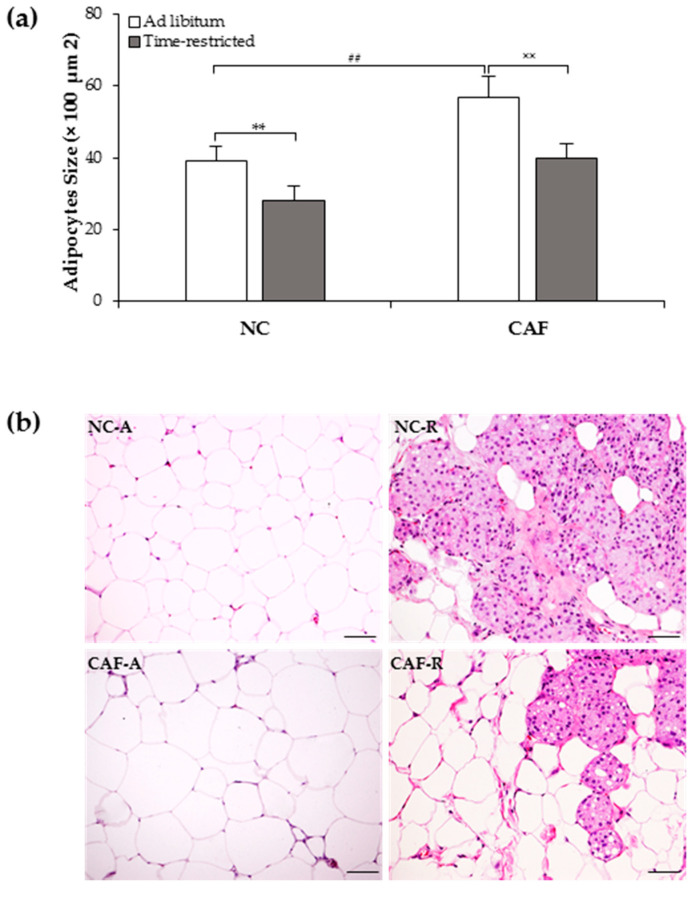
Effect of time-restricted feeding on inguinal WAT morphology of normal-chow- and cafeteria-fed rats: (**a**) mean adipocyte area was measured using a morphometric quantitative method at ×200 magnification with Axio Vision software; (**b**) representative hematoxylin and eosin staining of inguinal WAT sections (original magnification × 400): time-restricted feeding regimen induced the appearance of clusters of multilocular brown-like fat cells in normal-chow- and cafeteria-fed rats. NC: normal chow diet; CAF: cafeteria dietNC–A: normal chow ad libfeeding; NC–R: normal chow time-restricted feeding; CAF–A: cafeteria diet ad libfeeding; CAF–R: cafeteria time-restricted feeding. Values are means ± S.D (*n* = 200 adipocytes/group). ^##^ *p <* 0.01 CAF–A vs. NC–A rats; ** *p <* 0.01 NC–R vs. NC–A rats; ^××^ *p <* 0.01 CAF–R vs. CAF–A rats (Mann–Whitney U-test). Scale bar: 50 µm.

To determine the effect of TRF on morphological changes and browning induction in iWAT, histological analyses were performed in the inguinal depots. These depots were selected because they are those with a greater browning capacity, according to the literature [42]. Our results showed that adipocyte sizes were significantly higher in the CAF–A group compared to the NC–A group (57 ± 5.6 µm^2^ × 100 vs. 39 ± 4.2 µm^2^ × 100; *p* < 0.01; Figure 3a), and that the TRF paradigm decreased adipocytes size inrats fed with both NC-(28 ± 4.0 µm^2^ × 100) and CAF-diets (40 ± 4.1 µm^2^ × 100) as compared to their diet-matched ad librats (*p <* 0.01; Figure 3a). Interestingly, adipocyte size in CAF–R group was decreased to nearly that inthe NC–A group.

As shown in H&E-stained preparation (Figure 3b), the majority of cells in all groups showed an appearance of white unilocular adipocytes with a single lipid droplet. The TRF schedule induced the formation of multilocular brown-like adipocytes dispersed among unilocular adipocytes in both NC- and CAF-fed rats. The percentage of multilocularity represented around 31.1% and 15.2% of total area analyzed in the NC–R and CAF–R group, respectively.

### 3.4. Effects of TRF on UCP1 and PGC1α Contents

**Figure 4:**
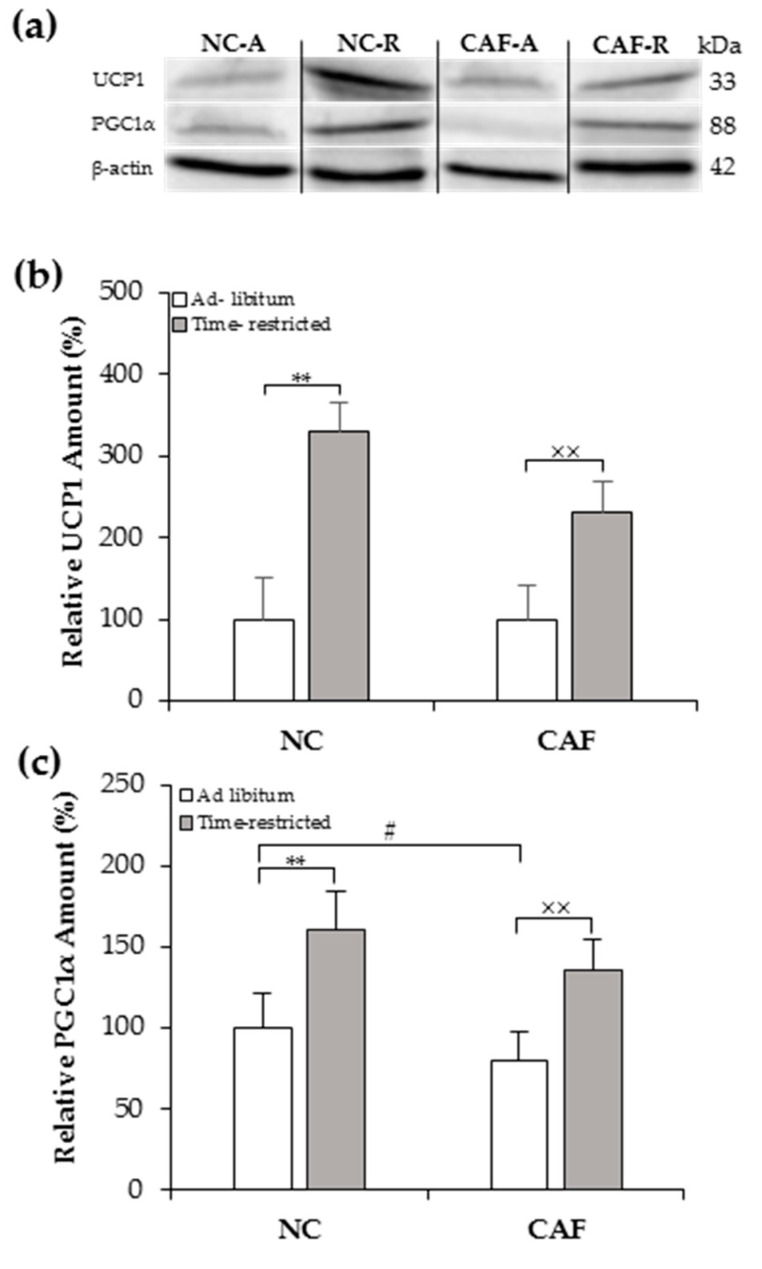
Effects of time-restricted feeding on thermogenic protein levels in the inguinal WAT of normal chow and cafeteria fed rats as measured by Western blot: (**a**) representative blot of UCP1 and PGC1α; (**b**,**c**) densitometry quantification of UCP1 and PGC1α protein levels. NC–A: normal chow ad libfeeding; NC–R: normal chow time-restricted feeding; CAF–A: cafeteria diet ad libfeeding; CAF–R: cafeteria time-restricted feeding; NC: normal chow diet; CAF: cafeteria diet.Values are means ± S.D (*n* = 3) of ratios of specific protein levels to β-actin (Loading protein). Data of the NC–A group wereset to 100% and the rest of the values are referred to this. ^#^ *p <* 0.05 CAF–A vs. NC–A rats; ** *p <* 0.01 NC–R vs. NC–A rats; ^××^ *p <* 0.01 CAF–R vs. CAF–A rats (Mann–Whitney U-test).

The molecular signature that identifies brown and brown-like adipocytes is UCP1 [43]. Therefore, we examined UCP1 protein levels in inguinal fat depots from both NC- and CAF-fed rats on TRF using Western blot. As expected from their microscopic aspect, UCP1 protein expression was significantly enhanced in the inguinal fat of NC–R and CAF–R groups by ~3 and ~2 fold, respectively, as compared to their diet-matched ad lib groups (*p <* 0.01; Figure 4b). Importantly, a weak signal of UCP1 was observed in the inguinal fat ofad lib-fed rats, whereas TRF rats exhibited a more pronounced UCP1 signal (Figure 4a).

PCG1α is a master nuclear transcription factor that controls the expression of the thermogenic gene program, including the expression of the UCP1 gene. Its expression level was significantly enhanced in inguinal fat depots of the NC–R and CAF–R groups by ~1.5 fold, as compared to their ad lib counterparts (*p <* 0.01; Figure 4c).

## 4. Discussion

Herein, we report, for the first time, that a TRF regimen prevents obesity and associated dyslipidemia in CAF-diet-induced obese rat model. This effect occurred probably via browning of the iWATwithout changing the calorie intake or locomotor activity.

Our results showed that consumption of calorically dense, palatable human foods induced an excessive caloric intake, resultingin rapid and drastic weight gain, abdominal adiposity, and dyslipidemic state in thead lib-fed group, suggesting that the CAF-diet used in the present study successfully induced an animal model of obesity. As shown in Table S1, the CAFdiet contained foods that are high in fat and saturated fatty acids; hence, the weight gain in the CAF-ad lib group could be due to the high rate of acylation of saturated fatty acids into triglycerides that are subsequently stored in the adipose tissue [44]. Besides, post-ingestive effects of high-fat food contribute to weight gain through reduction of satiety signals [45] and attenuation of fatty acid oxidation [46]. Interestingly, TRF significantly reduced the body weight gain in our rats fed on a CAF-diet, suggesting thatTRF is an effective strategy for weight reduction with aCAF-style diet that models the Western pattern diet. Similar findings have also been previously reported in different animal strains after exposure to TRF of other obesogenic diets [8,9,12,19,20,21,23]. This has also been observed among some overweight or obese humans undergoing a TRF eating pattern [14,15]. It should be noticed that the body weight gain-lowering effect of TRF in the current study took place in the absence of changes in caloric intake and locomotor activity, which is consistent with previous studies [8,9,12,19]. Therefore, the mechanism whereby TRF reduced body weight cannot simply be explained by changes in quantity of diet or physical activity, which represent the locomotor activity of 5 min duration of the dark phase (phase of activity) of the 15th week of the experiment, but rather by other mechanisms, as discussed below. Our present data of calorie intake are not consistent with another previous report in which mice fed an HFD under TRF of 12 h during the dark or light phase were unable to fully compensate for the limited availability of food, and calorie intake was significantly reduced compared with their ad lib counterparts [47]. The reason for this discrepancy is uncertain and requires further investigation.

One potential explanation to account for the attenuated body weight gain without any overall change in calorie intake or physical activity upon TRF is through switching lipid metabolism from storage to oxidative as a strategy to enhance energy expenditure. Although energy metabolism was not measured in the current study, the study by Panda’s group showed that 8hTRF during the active phase attenuated the HFD-induced dampening of the daily rhythm of the respiratory exchange ratio and led to an overall increase in oxygen consumption and energy expenditure in mice, indicating lipid utilization for energy metabolism [8]. In another study by the same group, TRF of HFD or normocaloric diet increased PGC1α gene expression in WAT of mice, leading to increased β-oxidation [9], which corroborates our data of PGC1α.

Ourfindingsconcerning body weight gain paralleled those of fat accumulation measurements. Notably, rats fed a CAFdietad lib exhibited drastic increases in fat pad weights (inguinal, mesenteric, epididymal, retroperitoneal) compared to rats fed an NCdietad lib. These observations suggest that the exposure of rats to calorically dense diets facilitated fat accumulation in the abdominal regions due to the high effective energy content of the high-fat foods [48]. Previous studies have observed a marked reduction in adipose tissue mass in rodents under a TRF regimen [8,9,23], which is in agreement with the present finding showing that TRF of NC- or CAF-diet reduced fat accumulation in subcutaneous and visceral fat, resulting in a significant decrease in total adiposity. The reduction of fat accumulation in WAT under TRF may be due to an overall increase in fatty acid oxidation and a decrease in free fatty acid synthesis [9], which paralleledthe current data showing that adipocytes size of iWATinTRF rats were smaller than thatin diet-matched ad lib rats.

The excessive weight gain in the CAF–Agroupwas associated with a dyslipidemic condition, as evidenced by abnormally elevated serum levels of TG, TC, and LDL-c and reduced serum level of HDL-c, which is consistent with the findings of a previous study using a CAFdiet [49]. The dyslipidemia was probably evident due to the overall higher consumption of the CAFdiet, which, while being high in fat, is also high in saturated fatty acids, known to increase the production of TG and LDL-c by the liver [50], and in cholesterol, which can result in a hypercholesterolemia [51] and reduced HDL production or HDL clearance from plasma [52]. Dyslipidemia may also beexplained bya hepatic lipid accumulation, which was associated with disrupted circadian rhythms and food intake patterns in response to high caloric diet [53]. Interestingly, the TRF regimen improved the negative lipid profile in CAF-fed rats by decreasing serum levels of TC, TG, and LDL-c and increasing serum level of HDL-c, suggesting its anti-hyperlipidemic effect.However, TRFunexpectedly had no effect on dyslipidemic parameters when rats were fed with anNCdiet, in spite of a marked effect on body weight. The lack of the TRF effect on dyslipidemic parameters in normocaloric-fed rats may indicate that the influence of TRF may be negligible when the overall content of diet is less caloric, which would require future investigation.To our knowledge, only three studies have been reported so far on the anti-hyperlipidemic potential of TRF in DIO models(Chaix et al. [9], Sherman et al. [23], and Sun et al. [12]). It is important to note that there were considerable variations in experimental design, such as obesogenic diets, animal strain, feeding window, and experimental durations between the present study and the aforementioned studies, which make direct comparison difficult.The mechanism wherebyTRF underlies lipid parameters is unclear. Typically, weight loss is expected to promote lipidimprovements [54]; however, outcomes from thecurrent studydo not consistently support improvements in body weight as, despite reduced body weight gain, no changes in lipid panel were recorded in NC-fed rats. Since the liver is a central organ in lipid metabolism, we suggest that the beneficial effect of a TRF regimen on lipid profiles in CAF-fed ratsmay be due to the enhancement of hepatic lipid metabolism. This is supported by several studies in which TRF has been found to enhance lipid oxidation over lipogenesis [8,12,21,55]. Indeed, TRF has been shown to reduce hepatic expression of PPARγ and SREBF1, the key lipogenic genes, in HFD-fed rats [55]. In addition, TRF regimen reduced lipid droplet associated and lipolysis inhibitor gene Cidec expressions, TG storage-associated protein CD36, and plasma TG marker ApoA 4 in HFD-fed mice [8].Decreased TG in the liver results in reduced serum levels of LDL and very-low-density lipoprotein (VLDL), leading to loss of transported cholesterol and TG within them [54]. The TC-lowering effect of TRF may also be attributed to enhanced cholesterol catabolism to bile acids biosynthesis in theliver, as indicated by increased expression of two rate-limiting enzymes of bile acids biosynthesis, namelyHmgcs 2 and Cyp7α1, in the liver of time-restricted mice [9].

It is commonly thought that unfavorable lipid profiles, synonymous with intake of dietary saturated fat, are the hallmark for the progression of cardiovascular diseases (CVD) and coronary heart diseases [56].Observations from epidemiological studies have confirmed that high TC, LDL-c, and TG and low HDL-c levels are associated with increased risk of CVD [56,57,58,59]. Together, thesedatacorroborate the present findings showing marked increases in AIP, AC, and CRI in CAF–A rats compared to NC–A rats. These indices are strong indicators of the CVD risk in clinical practices by their expression of imbalance between atherogenic and anti-atherogenic lipoproteins [60]. A recent human pilot study reported the effectiveness of 10 h time-restricted eating intervention for 12 wks to lower atherogenic lipids in obese subjects with metabolic syndrome, leading to improved cardiometabolic health [61]. Herein, TRF reduced the calculated values of AIP, AC, and CRIin CAF-fed rats, suggesting its very interesting cardioprotective potential. Since this is the first study to examine the effect of TRF onatherogenic indices in an animal model of obesity, these results should be considered preliminary; therefore, further in-depth investigationsare warranted to confirm the cardioprotective properties of TRF intervention in DIO models.

Since white fat browning is associated with body weight loss and metabolic improvements, we aimed to characterize the morphological and molecular features of iWAT. As hypothesized, we found that, in TRF rats, there was an obvious appearance of multilocular brown adipocytes and increased UCP1 and PGC1α protein expressions in iWAT of NC- and CAF-fed rats, indicating its potential to promote WAT browning.In one interesting study, intermittent fasting, an eating pattern that involves a 24 h fasting period on 2–3 days per week, or on alternate days, was reported to promote inguinal and gonadal fat browning in lean and obese mice [62]. In support of these findings, results from the current study showed that 8hTRF induced iWAT browning in normocaloric and CAF-fed rats. To the best of our knowledge, this report is the first to show the potential of TRF to promote WAT browning.Direct experimental evidence for the role of UCP1 in counteracting obesity has been reported previously in many studies. Of note, an animal model of genetic obesity using adipose tissue-targeted overexpression of UCP1 resulted in a reduced level of obesity [63]. In addition, UCP1 ablation resulted in increased obesity and metabolic deficiency in obesity-resistance mouse strain [64]. In humans, UCP1 gene polymorphism was significantly associated with increased body mass index in obese subjects [65]. Therefore, the present data concerning the iWAT browning and UCP1 expressionmay decipher the possible mechanism behind the body weight gain-lowering effect of a TRF regimen. Although the current data support the possibility that a TRF regimen could act as a WAT browning inducer, further in-depth studies are warranted to understand whether a TRF regimen could promote browningin other WAT depots and to investigate the expression of browning-related genes. Furthermore, the present study is limited by the fact that we did not measure whole-body energy expenditure due to practical reasons; hence, to what extent the effects of a TRF regimen on WAT browning andUCP1 content could be translated into increased whole-body energy expenditure is unclear, and future studies will address this shortcoming.A further limitation is that we did not measure a 24-h profile of locomotor activity. Therefore, the 5-min locomotor activity, which was measured at different times between groups, could induce time effects rather than TRF effects. However, as the locomotor activity for all groups was assessed within a short period of 2 h during the dark phase, it suggests that the locomotor activity was not affected by different times between groups but rather by TRF, although this hypothesis needs to be confirmed.

## 5. Conclusions

This present study showed that TRF regimen improved body weight gain and dyslipidemia in CAF-diet model and acted as white fat browning inducer with thermogenic properties, which may explain the anti-obesity effect of TRF.

## Figures and Tables

**Table 1:** Effects of time-restricted feeding on relative white fat pads weightand total adiposity.

Parameters	NC–A	NC–R	CAF–A	CAF–R
iWAT (g/100 g b.w)	1.1 ± 0.3	0.9 ± 0.3 *	2.4 ± 0.3 ^##^	1.1 ± 0.3 ^××^
mWAT (g/100 g b.w)	0.9 ± 0.2	0.4 ± 0.2 **	3.0 ± 0.3 ^##^	1.0 ± 0.4 ^××^
eWAT (g/100 g b.w)	1.2 ± 0.3	0.5 ± 0.2 **	2.9 ± 0.3 ^##^	1.3 ± 0.4 ^××^
rWAT (g/100 g b.w)	1.0 ± 0.2	0.6 ± 0.2 *	2.4 ± 0.4 ^##^	1.2 ± 0.4 ^××^
Total VAT (g/100 g b.w)	3.1 ± 0.8	1.5 ± 0.4 **	8.2 ± 1.0 ^##^	3.5 ± 0.6 ^××^
Total Adiposity (%)	4.2 ± 0.7	2.5 ± 0.6 **	10.6 ± 1.0 ^##^	4.7 ± 1.0 ^××^

NC–A: normal chow ad libfeeding; NC–R: normal chow time-restricted feeding; CAF–A: cafeteria diet ad libfeeding; CAF–R: cafeteria time-restricted feeding; WAT: white adipose tissue; iWAT: inguinal WAT; mWAT: mesenteric WAT; eWAT: epididymal WAT; rWAT: retroperitoneal WAT; VAT: visceral adipose tissue; b.w: body weight. Values are means ± S.D (*n* = 6). ^##^ *p* < 0.01 CAF–A vs. NC–A rats; * *p* < 0.05, ** *p* < 0.01 NC–R vs. NC–A rats; ^××^ *p* < 0.01 CAF–R vs. CAF–A rats (Mann–Whitney U-test).

**Table 2:** Effects of time-restricted feeding on serum lipid profiles and atherogenic indices.

Parameters	NC–A	NC–R	CAF–A	CAF–R
		Serum lipid profiles		
TG (mg/dL)	75.1 ± 5.4	71.2 ± 5.1	101.1 ± 3.9 ^##^	81.0 ± 4.2 ^××^
TC (mg/dL)	64.5 ± 6.9	63.7 ± 6.6	93.8 ± 4.4 ^##^	68.0 ± 5.6 ^××^
HDL-c (mg/dL)	54.0 ± 7.4	55.2 ± 4.9	38.7 ± 5.6 ^##^	50.3 ± 5.1 ^××^
LDL-c (mg/dL)	18.6 ± 4.7	15.9 ± 3.4	35.3 ± 3.7 ^##^	19.8 ± 4.7 ^××^
		Atherogenic indices		
AIP	0.2 ± 0.1	0.1 ± 0.1	0.4 ± 0.1 ^##^	0.2 ± 0.1 ^××^
AC	0.2 ± 0.3	0.2 ± 0.3	1.5 ± 0.3 ^##^	0.4 ± 0.1 ^××^
CRI	1.2 ± 0.6	1.1 ± 0.2	2.4 ± 0.8 ^##^	1.3 ± 0.6 ^××^

NC–A: normal chow ad libfeeding; NC–R: normal chow time-restricted feeding; CAF–A: cafeteria diet ad libfeeding; CAF–R: cafeteria time-restricted feeding; TG: total triglyceride; TC: total cholesterol; HDL-c: high density lipoprotein cholesterol; LDL-c: low density lipoprotein cholesterol; AIP: atherogenic index of plasma; AC: atherogenic coefficient; CRI: coronary risk index. Values are means ± S.D (*n* = 6). ^##^ *p <* 0.01 CAF–A vs. NC–A rats; ^××^ *p <* 0.01 CAF–R vs. CAF–A rats (Mann–Whitney U-test).

## Supplementary Materials

The following are available online at https://www.mdpi.com/2072-6643/12/8/2185/s1, Table S1: normal chow and cafeteria food items compositions as given by the product manufacturers.

## References

[1] WHO (2018). Overweight and Obesity.

[2] Bouchard C. (2008). Gene-environment interactions in the etiology of obesity: Defining the fundamentals. Obesity.

[3] Brantley P., Myers V.H., Roy H.J. (2005). Environmental and Lifestyle Influences on Obesity. J. La State Med. Soc..

[4] Procaccini C., Carbone F., Galgani M., La Rocca C., De Rosa V., Cassano S., Matarese G. (2011). Obesity and susceptibility to autoimmune diseases. Expert Rev. Clin. Immunol..

[5] Hotamisligil G.S. (2006). Inflammation and metabolic disorders. Nature.

[6] Anderson J.W., Konz E.C., Frederich R.C., Wood C.L. (2001). Long-term weight-loss maintenance: A meta-analysis of US studies. Am. J. Clin. Nutr..

[7] Longo V.D., Panda S. (2016). Fasting, Circadian Rhythms, and Time-Restricted Feeding in Healthy Lifespan. Cell Metab..

[8] Hatori M., Vollmers C., Zarrinpar A., DiTacchio L., Bushong E.A., Gill S., Leblanc M., Chaix A., Joens M., Fitzpatrick J.A.J. (2012). Time-restricted feeding without reducing caloric intake prevents metabolic diseases in mice fed a high-fat diet. Cell Metab..

[9] Chaix A., Zarrinpar A., Miu P., Panda S. (2014). Time-restricted feeding is a preventative and therapeutic intervention against diverse nutritional challenges. Cell Metab..

[10] Olsen M.K., Choi M.H., Kulseng B., Zhao C.M., Chen D. (2017). Time-restricted feeding on weekdays restricts weight gain: A study using rat models of high-fat diet-induced obesity. Physiol. Behav..

[11] Cote I., Toklu H.Z., Green S.M., Morgan D., Carter C.S., Tümer N., Scarpace P.J. (2018). Limiting feeding to the active phase reduces blood pressure without the necessity of caloric reduction or fat mass loss. Am. J. Physiol. Integr. Comp. Physiol..

[12] Sun S., Hanzawa F., Umeki M., Ikeda S., Mochizuki S., Oda H. (2018). Time-restricted feeding suppresses excess sucrose-induced plasma and liver lipid accumulation in rats. PLoS ONE.

[13] Sutton E.F., Beyl R., Early K.S., Cefalu W.T., Ravussin E., Peterson C.M. (2018). Early Time-Restricted Feeding Improves Insulin Sensitivity, Blood Pressure, and Oxidative Stress Even without Weight Loss in Men with Prediabetes. Cell Metab..

[14] Gabel K., Hoddy K.K., Haggerty N., Song J., Kroeger C.M., Trepanowski J.F., Panda S., Varady K.A. (2018). Effects of 8-hour time restricted feeding on body weight and metabolic disease risk factors in obese adults: A pilot study. Nutr. Healthy Aging.

[15] Antoni R., Robertson T.M., Robertson M.D., Johnston J.D. (2018). Metabolic physiology in free-living human subjects. J. Nutr. Sci..

[16] Moro T., Tinsley G., Bianco A., Marcolin G., Pacelli Q.F., Battaglia G., Palma A., Gentil P., Neri M., Paoli A. (2016). Effects of eight weeks of time-restricted feeding (16/8) on basal metabolism, maximal strength, body composition, inflammation, and cardiovascular risk factors in resistance-trained males. J. Transl. Med..

[17] Gill S., Panda S. (2015). A Smartphone App Reveals Erratic Diurnal Eating Patterns in Humans that Can Be Modulated for Health Benefits. Cell Metab..

[18] Delahaye L.B., Bloomer R.J., Butawan M.B., Wyman J.M., Hill J.L., Lee H.W., Liu A.C., McAllan L., Han J.C., Van Der Merwe M. (2018). Time-restricted feeding of a high-fat diet in male C57BL/6 mice reduces adiposity but does not protect against increased systemic inflammation. Appl. Physiol. Nutr. Metab..

[19] Duncan M.J., Smith J.T., Narbaiza J., Mueez F., Bustle L.B., Qureshi S., Fieseler C., Legan S.J. (2016). Restricting feeding to the active phase in middle-aged mice attenuates adverse metabolic effects of a high-fat diet. Physiol. Behav..

[20] Sundaram S., Yan L. (2016). Time-restricted feeding reduces adiposity in mice fed a high-fat diet. Nutr. Res..

[21] Chung H., Chou W., Sears D.D., Patterson R.E., Webster N.J.G., Ellies L.G. (2016). Time-restricted feeding improves insulin resistance and hepatic steatosis in a mouse model of postmenopausal obesity. Metabolism.

[22] Park S., Yoo K.M., Hyun J.S., Kang S. (2017). Intermittent fasting reduces body fat but exacerbates hepatic insulin resistance in young rats regardless of high protein and fat diets. J. Nutr. Biochem..

[23] Sherman H., Genzer Y., Cohen R., Chapnik N., Madar Z., Froy O. (2012). Timed high-fat diet resets circadian metabolism and prevents obesity. FASEB J..

[24] Chaix A., Lin T., Le H.D., Chang M.W., Panda S. (2019). Time-Restricted Feeding Prevents Obesity and Metabolic Syndrome in Mice Lacking a Circadian Clock. Cell Metab..

[25] Sherman H., Frumin I., Gutman R., Chapnik N., Lorentz A., Meylan J., le Coutre J., Froy O. (2011). Long-term restricted feeding alters circadian expression and reduces the level of inflammatory and disease markers. J. Cell. Mol. Med..

[26] Buettner R., Schölmerich J., Bollheimer L.C. (2007). High-fat diets: Modeling the metabolic disorders of human obesity in rodents. Obesity.

[27] Nilsson C., Raun K., Yan F.F., Larsen M.O., Tang-Christensen M. (2012). Laboratory animals as surrogate models of human obesity. Acta Pharmacol. Sin..

[28] Sampey B.P., Vanhoose A.M., Winfield H.M., Freemerman A.J., Muehlbauer M.J., Fueger P.T., Newgard C.B., Makowski L. (2011). Cafeteria diet is a robust model of human metabolic syndrome with liver and adipose inflammation: Comparison to high-fat diet. Obesity.

[29] Gomez-Smith M., Karthikeyan S., Jeffers M.S., Janik R., Thomason L.A., Stefanovic B., Corbett D. (2016). A physiological characterization of the Cafeteria diet model of metabolic syndrome in the rat. Physiol. Behav..

[30] Lewis A.R., Singh S., Youssef F.F. (2019). Cafeteria-diet induced obesity results in impaired cognitive functioning in a rodent model. Heliyon.

[31] Shafat A., Murray B., Rumsey D. (2009). Energy density in cafeteria diet induced hyperphagia in the rat. Appetite.

[32] Panda S. (2016). Circadian physiology of metabolism. Science.

[33] Chaix A., Zarrinpar A. (2015). The effects of time-restricted feeding on lipid metabolism and adiposity. Adipocyte.

[34] Harms M., Seale P. (2013). Brown and beige fat: Development, function and therapeutic potential. Nat. Med..

[35] Jiménez-Aranda A., Fernández-Vázquez G., Campos D., Tassi M., Velasco-Perez L., Tan D.X., Reiter R.J., Agil A. (2013). Melatonin induces browning of inguinal white adipose tissue in Zucker diabetic fatty rats. J. Pineal Res..

[36] Sestakova N., Puzserova A., Kluknavsky M., Bernatova I. (2013). Determination of motor activity and anxiety-related behaviour in rodents: Methodological aspects and role of nitric oxide. Interdiscip. Toxicol..

[37] Chaix A., Manoogian E.N.C., Melkani G.C., Panda S. (2019). Time-Restricted Eating to Prevent and Manage Chronic Metabolic Diseases. Annu. Rev. Nutr..

[38] Fan S.J., Jiang H., Yang L.J., Liu X., Song J., Pan F. (2011). Effects of adrenergic agents on stress-induced brain microstructural and immunochemical changes in adult male Wistar rats. Ann. Anat..

[39] Althunibat O.Y., Al Hroob A.M., Abukhalil M.H., Germoush M.O., Bin-Jumah M., Mahmoud A.M. (2019). Fisetin ameliorates oxidative stress, inflammation and apoptosis in diabetic cardiomyopathy. Life Sci..

[40] Adedokun A.K., Olisekodiaka M.J., Adeyeye D.A., Muhibi A.M., Ojokuku O.H., Adepeju A.A., Onifade A.A., Adetoro A.T., Ajibola A.K., Sheu M.R. (2017). Castelli Risk Index, Atherogenic Index of Plasma, and Atherogenic Coefficient: Emerging Risk Predictors of Cardiovascular Disease in HIV-Treated Patients. Saudi J. Med. Pharm. Sci..

[41] Seki M., Ishiguro T., Gyohda Y., Ohsato S., Yokota M. (1998). Evaluation of the Efficacy of Coronary Risk Index, a New Scoring System for Predicting Morbidity and Severity of Coronary Stenosis in Patients Undergoing Coronary Angiography. J. Jpn. Soc. Clin. Anesth..

[42] Zuriaga M.A., Fuster J.J., Gokce N., Walsh K. (2017). Humans and Mice Display Opposing Patterns of “Browning” Gene Expression in Visceral and Subcutaneous White Adipose Tissue Depots. Front. Cardiovasc. Med..

[43] Liu X. (2017). Common and distinct regulation of human and mouse brown and beige adipose tissues: A promising therapeutic target for obesity. Protein Cell.

[44] Storlien L.H., Huang X.F., Lin S., Xin X., Wang H.Q., Else P.L. (2000). Dietary Fat Subtypes and Obesity. In Fatty Acids and Lipids. New Find..

[45] Kentish S., Li H., Philp L.K., O’Donnell T.A., Isaacs N.J., Young R.L., Wittert G.A., Blackshaw L.A., Page A.J. (2012). Diet-induced adaptation of vagal afferent function. J. Physiol..

[46] Little T.J., Horowitz M., Feinle-Bisset C. (2007). Modulation by high-fat diets of gastrointestinal function and hormones associated with the regulation of energy intake: Implications for the pathophysiology of obesity. Am. J. Clin. Nutr..

[47] Kentish S.J., Hatzinikolas G., Li H., Frisby C.L., Wittert G.A., Page A.J. (2018). Time-restricted feeding prevents ablation of diurnal rhythms in gastric vagal afferent mechanosensitivity observed in high-fat diet-induced obese mice. J. Neurosci..

[48] DeLany J.P., Windhauser M.M., Champagne C.M., Bray G.A. (2000). Differential Oxidation of Individual Dietary Fatty Acids in Humans. Am. J. Clin. Nutr..

[49] MacEdo I.C., Medeiros L.F., Oliveira C., Oliveira C.M., Rozisky J.R., Scarabelot V.L., Souza A., Silva F.R., Santos V.S., Cioato S.G. (2012). Cafeteria diet-induced obesity plus chronic stress alter serum leptin levels. Peptides.

[50] RJ N. (1997). Dietary Fat Saturation Effects on Low-Density-Lipoprotein Concentrations and Metabolism in Various Animal Models. Am. J. Clin. Nutr..

[51] Zulet M.A., Barber A., Martínez J.A., Garcin H., Higueret P. (1999). Alterations in Carbohydrate and Lipid Metabolism Induced by a Diet Rich in Coconut Oil and Cholesterol in a Rat Model. J. Am. Coll. Nutr..

[52] Thomas M.S., Prack M.M., Dashti N., Johnson F., Rudel L.L., Williams D.L. (1989). Differential Effects of Dietary Fat on the Tissue-Specific Expression of the Apolipoprotein A-I Gene: Relationship to Plasma Concentration of High Density Lipoproteins. J. Lipid Res..

[53] Christie S., Vincent A.D., Li H., Frisby C.L., Kentish S.J., O’rielly R., Wittert G.A., Page A.J. (2018). A rotating light cycle promotes weight gain and hepatic lipid storage in mice. Am. J. Physiol. Gastrointest. Liver Physiol..

[54] Santos H.O., Macedo R.C.O. (2018). Impact of intermittent fasting on the lipid profile: Assessment associated with diet and weight loss. Clin. Nutr. ESPEN.

[55] Woodie L.N., Luo Y., Wayne M.J., Graff E.C., Ahmed B., O’Neill A.M., Greene M.W. (2018). Restricted feeding for 9 h in the active period partially abrogates the detrimental metabolic effects of a Western diet with liquid sugar consumption in mice. Metabolism.

[56] Jellinger P.S., Smith D.A., Mehta A.E., Ganda O., Handelsman Y., Rodbard H.W., Shepherd M.D., Seibel J.A. (2012). American association of clinical endocrinologists’ guidelines for management of dyslipidemia and prevention of atherosclerosis. Endocr. Pract..

[57] Graham I., Cooney M.T., Bradley D., Dudina A., Reiner Z. (2012). Dyslipidemias in the prevention of cardiovascular disease: Risks and causality. Curr. Cardiol. Rep..

[58] Wilson P.W.F., D’Agostino R.B., Levy D., Belanger A.M., Silbershatz H., Kannel W.B. (1998). Prediction of Coronary Heart Disease Using Risk Factor Categories. Circulation.

[59] Boullart A.C.I., De Graaf J., Stalenhoef A.F. (2012). Serum triglycerides and risk of cardiovascular disease. Biochim. Biophys. Acta Mol. Cell Biol. Lipids.

[60] Millán J., Pintó X., Muñoz A., Zúñiga M., Rubiés-Prat J., Pallardo L.F., Masana L., Mangas A., Hernández-Mijares A., González-Santos P. (2009). Lipoprotein ratios: Physiological significance and clinical usefulness in cardiovascular prevention. Vasc. Health Risk Manag..

[61] Wilkinson M.J., Manoogian E.N.C., Zadourian A., Lo H., Fakhouri S., Shoghi A., Wang X., Fleischer J.G., Navlakha S., Panda S. (2020). Ten-Hour Time-Restricted Eating Reduces Weight, Blood Pressure, and Atherogenic Lipids in Patients with Metabolic Syndrome. Cell Metab..

[62] Liu B., Page A.J., Hutchison A.T., Wittert G.A., Heilbronn L.K. (2019). Intermittent fasting increases energy expenditure and promotes adipose tissue browning in mice. Nutrition.

[63] Kopecky J., Clarke G., Enerbäck S., Spiegelman B., Kozak L.P. (1995). Expression of the mitochondrial uncoupling protein gene from the aP2 gene promoter prevents genetic obesity. J. Clin. Investig..

[64] Luijten I.H.N., Feldmann H.M., von Essen G., Cannon B., Nedergaard J. (2019). In the absence of UCP1-mediated diet-induced thermogenesis, obesity is augmented even in the obesity-resistant 129S mouse strain. Am. J. Physiol. Endocrinol. Metab..

[65] Chathoth S., Ismail M.H., Vatte C., Cyrus C., Al Ali Z., Ahmed K.A., Acharya S., Al Barqi A.M., Al Ali A. (2018). Association of Uncoupling Protein 1 (UCP1) gene polymorphism with obesity: A case-control study. BMC Med. Genet..

