# Supplementary material for: Time-Restricted Feeding Improves Body Weight Gain, Lipid Profiles, and Atherogenic Indices in Cafeteria-Diet-Fed Rats: Role of Browning of Inguinal White Adipose Tissue
Source: Nutrients. 2020 Jul 23;12(8):2185. doi: 10.3390/nu12082185 (PMC7469029; doi:10.3390/nu12082185)
Supplement: Supplementary file 1 [file nutrients-12-02185-s001.pdf]

Table S1. Normal chow and cafeteria food items compositions as given by the product manufacturers.

**Values are Per 1g of Food Item**	Total (kcal)	Total Fat (g)	Saturated Fat (g)	Cholesterol (mg)	Protein (g)	Total Carb (g)	Sugars (g)	Dietary Fiber (g)	Sodium (mg)
Normal chow	3.10	0.06	0.00	0.00	0.19	0.44	0.04	0.03	
Cereals Lion Caramel Chocolat (Nestle)	4.06	0.07	0.03	0.00	0.08	0.76	0.29	0.05	2.00
Nesquick Cereals (Nestle)	3.96	0.02	0.01	0.00	0.08	0.76	0.25	0.09	4.40
Chocolate Chip Cookies (Bimo)	5.40	0.25	0.12	0.00	0.06	0.61	0.31	0.02	2.97
Cream Filled cookies (Dream)	4.92	0.24	0.14	0.00	0.05	0.65	0.27	0.03	1.46
Bar Peanuts Chocolat (Maxon)	5.65	0.37	0.14	0.15	0.05	0.55	0.40	0.00	1.32
Chocolat Filled with Caramel Cream (Bimo)	4.73	0.22	0.14	0.00	0.08	0.62	0.53	0.01	2.00
Mallow Pie (Alpella)	4.48	0.18	0.11	0.00	0.05	0.67	0.45	0.03	2.61
Mini Muffin (Dulcesol)	4.72	0.28	0.03	0.76	0.05	0.50	0.35	0.01	2.90
Snack' Choc Caramel (Alpella)	4.56	0.17	0.13	0.00	0.04	0.71	0.60	0.01	1.67
Truffle Snack (Dulcesol)	5.31	0.26	0.13	0.00	0.04	0.63	0.44	0.02	1.00
Crackers Cheese Flavored (Tuc)	4.85	0.23	0.10	0.06	0.08	0.61	0.05	0.02	10.60
Peanuts Corn Chips (Mr. Chips)	5.00	0.26	0.04	0.00	0.13	0.56	0.02	0.04	7.56
Potato Crisps (Mister)	5.10	0.29	0.15	0.00	0.06	0.54	0.03	0.05	7.81
Cream Filled Wafers (Bimo)	5.24	0.28	0.20	0.10	0.06	0.60	0.31	0.06	0.58
Beef Salami (Bellat)	3.70	0.30	0.09	0.90	0.22	0.02	0.00	0.00	12.49
Pâté Pizza Flavored (Bellat)	3.39	0.28	0.10	2.48	0.15	0.03	0.00	0.00	6.90
Chicken Liver Pâté (Bellat)	2.13	0.14	0.06	3.89	0.14	0.06	0.00	0.00	3.80
Beef Corned (Bellat)	2.14	1.34	0.05	0.80	0.23	0.00	0.00	0.00	7.85
Cheese Cheddar (Chebli)	4.02	0.33	0.21	1.05	0.25	0.01	0.00	0.00	6.22
